# Results of radiation therapy combined with nedaplatin (cis-diammine-glycoplatinum) and 5-Fluorouracil for postoperative locoregional recurrent esophageal cancer

**DOI:** 10.1186/1471-2407-6-50

**Published:** 2006-03-04

**Authors:** Keiichi Jingu, Kenji Nemoto, Haruo Matsushita, Chiaki Takahashi, Yoshihiro Ogawa, Toshiyuki Sugawara, Eiko Nakata, Yoshihiro Takai, Shogo Yamada

**Affiliations:** 1 Department of Therapeutic Radiology, Tohoku University School of Medicine, Seiryo-machi 1-1, Aoba-ku, Sendai 980-8574, Japan

## Abstract

**Background:**

Although the effectiveness of radiotherapy with concurrent administration of several anti-tumor drugs for postoperative recurrent esophageal cancer has been demonstrated, the results are not satisfactory. The purpose of the present study was to evaluate the effectiveness and safety of radiotherapy combined with nedaplatin and 5-FU for postoperative locoregional (excluding hematogenous metastasis) recurrent esophageal cancer.

**Methods:**

In June 2000, we started a phase II study on treatment of postoperative locoregional recurrent esophageal cancer with radiotherapy (60 Gy/30 fr/6 weeks) combined with chemotherapy consisting of two cycles of nedaplatin (70 mg/m^2^/2 h) and 5-FU (500 mg/m^2^/24 h for 5 days).

The primary endpoint of the present study was overall survival rate, and the second endpoints were irradiated-field control rate, tumor response and toxicity.

**Results:**

A total of 30 patients were included in this study. The 1-year and 3-year overall survival rates were 60.6% and 56.3%, respectively, with a median survival period of 39.0 months, and the 1-year and 3-year irradiated-field control rates were 86.4% and 72%, respectively. Complete response and partial response were observed in 13.3% and 60.0% of the patients, respectively. Grade 3 or higher leukocytopenia and thrombocytopenia were observed in 30% and 3.3% of the patients, respectively, but renal toxicity of grade 3 or higher was not observed. The regimen was completed in 76.7% of the patients.

In univariate analysis, the difference between survival rate in preradiotherapy performance status, recurrent pattern (worse for patients with anastomotic recurrence) and age (worse for younger patients) were statistically significant.

**Conclusion:**

Radiotherapy combined with nedaplatin and 5-FU is a safe and effective salvage treatment for postoperative locoregional recurrent esophageal cancer.

## Background

Since the mid-1980's, extended radical esophagectomy with three-field (neck, mediastinum, and abdomen) lymph node dissection has been performed, and it seems to have improved survival of patients with esophageal cancer [1-3]. However, there is recurrence in 27~52% of operated patients and locoregional recurrence in 41.5~55% of patients with postoperative recurrence [3-9]. Although the effectiveness of radiotherapy and concurrent chemoradiotherapy using cisplatin (CDDP) + 5-fluorouracil (5-FU) or a combination of several anti-tumor drugs for postoperative recurrent esophageal cancer has been demonstrated, median survival periods have been only 7.0~11.0 months [9-19]. These results are not satisfactory.

**Figure 1:**
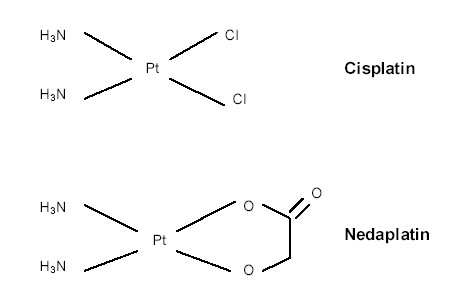
Chemical structures of cisplatin and nedaplatin.

Nedaplatin (Cis-Diammine-Glycoplatinum:CDGP), a derivate of CDDP that shows anti-tumor activity similar to that of CDDP and has less renal and gastrointestinal toxicity [20-23], is now being used clinically to treat cancer patients in Japan. The chemical structures of CDGP and CDDP are shown in Fig. 1. The rate of response to CDGP alone for treatment of esophageal cancer was reported to be 51.7% (15 partial responses obtained in 29 patients) [21]. CDGP + 5-FU seemed to have a superior effect to that of CDDP + 5-FU in a preclinical study [22] and has been shown to be safe and effective for treatment of esophageal cancer in some clinical studies [16,17,24,25].

Based on these facts, we started a phase study II on the effectiveness of radiotherapy combined with CDGP and 5-FU for postoperative locoregional recurrence of esophageal cancer.

## Methods

In June 2000, we started the present study in three institutes, Tohoku University Hospital and two affiliated hospitals, according to the following protocol.

All patients had histologically proven squamous cell carcinoma of the esophagus. Patient selection criteria included 1) 30 to 80 years of age, 2) Eastern Cooperative Oncology Group (ECOG) performance status of 0 to 3, 3) no other active cancer, 4) no serious cardiac, liver, or pulmonary disease, 5) creatinine clearance of more than 50 ml/min, 6) adequate bone marrow function (leukocyte count of 4000/μl, platelet count of 100,000/μl, 7) locoregional recurrence (including para-aortic lymph node metastasis) without distant metastasis after no residual tumor (R0) resection; extended radical esophagectomy with three-field (neck, mediastinum, and abdomen) lymph node dissection, and 8) no previous therapy other than R0 resection.

Recurrence was diagnosed comprehensively by upper gastrointestinal endoscopy, ultrasonography, computed tomography (CT), physical findings and/or cytology.

A linear accelerator (4 MV or 10 MV) was used as the X-ray source. The target volume was localized for radiotherapy in all patients by CT planning. The daily fractional dose of radiotherapy was 2.0 Gy, administered 5 days a week, and the total dose was 60.0 Gy. For 11 patients who had metastasis of lymph nodes in some regions or metastasis of many lymph nodes in one region, a T-shaped field (including the bilateral supraclavicular, mediastinal and abdominal regions) was used. For the remaining 19 patients, local fields with a margin of 1 to 2 cm from the macroscopic tumor were used. After a total dose of 40 Gy, the field was changed for all patients to avoid the spinal cord, and only macroscopic lesions were irradiated with a margin of 1 to 1.5 cm. To decrease the incidence of radiation pneumonitis, we avoided as much as possible irradiating more than 30% of V_20_, which is the percentage of the total lung volume that received > 20 Gy.

**Figure 2:**
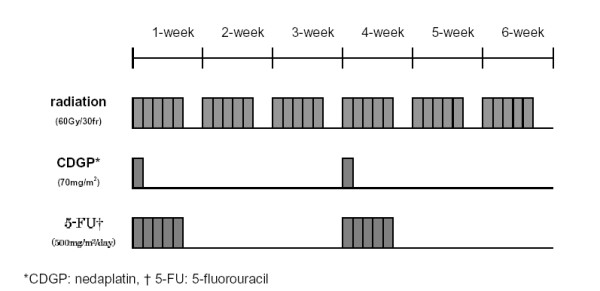
Schedule of the protocol of chemoradiotherapy.

Each cycle of chemotherapy consisted of 120-minute infusion of CDGP at 70 mg/m^2 ^and a 5-day period of 5-FU at 500 mg/m^2^/day. The median doses per body of CDGP and 5-FU were 100 mg/day (range, 80 to 125 mg/day) and 750 mg/day (range, 500 to 900 mg/day), respectively. This cycle of chemotherapy was repeated with an interval of 3 or 4 weeks, for a total radiotherapy dose of 60 Gy (Fig. 2). However, if toxicity of grade 3 or higher was noted and prolonged, we suspended or discontinued chemotherapy or reduced the dose of CDGP alone or the dose of both CDGP and 5-FU by 25~30% in the subsequent cycle.

Completion of the regimen in this study was defined as completion of two cycles of full-dose CDGP + 5-FU for a total radiotherapy dose of 60 Gy without suspension of treatment.

The overall survival, relapse-free survival and irradiated-field control rates were calculated from the first date of radiotherapy.

The primary endpoint of the current study was overall survival rate, and the second endpoints were tumor response, relapse-free survival rate, irradiated-field control rate and toxicity.

RECIST (Response Evaluation Criteria in Solid Tumors) was used to determine the tumor response. Tumor response was evaluated by CT 1~2 months after chemoradiotherapy. The number of mean measurable lesions was 1.8 per patient, and the response was evaluated according to agreement of more than two radiation-oncologists. In the present study, metastasis of para-aortic lymph nodes was defined as regional recurrence.

Follow-up evaluations were performed every 3~6 months for the first 2 years and every 12 months thereafter by endoscopy and CT.

We defined what only progression disease (PD) according was the failure of the present regimen (relapse again).

Survival estimates were calculated using the Kaplan-Meier method, and differences were evaluated by the log-rank test. Cox's proportional hazards regression model was used for univariate survival analysis. Age, preoperative stage (I – II vs. III – IV: Union International Contre le Cancer 1997 (UICC1997)), time interval between surgery and recurrence, pre-radiotherapy performance status (0–1 vs. 2–3), radiation field (local alone vs. T-shaped), acute tumor response (complete regression (CR) ~ partial regression (PR) vs. stable disease (SD) ~ PD), relapse again inside the irradiated field (yes vs. not), number of cycles of chemotherapy (one vs. two), recurrent pattern (anastomotic vs. non-anastomotic) and number of recurrent regions (one region vs. multiple regions) were entered into univariate analysis. In univariate analysis, age and time interval between surgery and recurrence were not classified in categories. A p value of less than 0.05 was considered significant. All analyses were performed using SPSS 11.0.

Toxicity was graded according to the Common Terminology Criteria for Adverse Events (CTCAE v3.0).

The present study protocol was reviewed and approved by the Tohoku University Hospital Institutional review board, and informed consent was obtained from each patient before conducting the treatment.

## Results

From June 2000 to December 2004, a total of 30 patients (29 males, 1 female; median age, 64 years; age range, 50 to 72 years) were enrolled in this phase II study. Patient characteristics are shown in Table 1. The sites of recurrence were supraclavicular lymph nodes (9 patients), mediastinal lymph nodes (14 patients), abdominal (including para-aortic) lymph nodes (7 patients) and anastomotic recurrence (9 patients). Five patients had recurrence or metastatsis in two regions and 2 patients had recurrence in three regions. The median time interval from surgery to recurrence was 12.5 months (range, 4 to 102 months). The median period of the regimen in the present study was 42 days (range, 37 to 106 days). Although all of the patients except for one patient who had a 59-day idle period because of acute cholecystitis completed the regimen of radiotherapy without suspension of treatment, 7 patients did not complete the regimen of chemotherapy because of adverse events in the acute phase (The second cycle of chemotherapy was cancelled in 5 patients, and the dose of CDGP alone or the dose of both CDGP and 5-FU were reduced in 2 patients.). The rate of completion of this regimen was 76.7%.

The last observation date was August 31, 2005. The median follow-up period was 12.5 months (range, 4.0 to 62.0 months) for all patients and 18.0 months (range, 4.5 to 62.0 months) for patients still alive. Sixteen of the 30 patients had relapse again. Thirteen patients out of a total of 30 died; 10 patients due to progression disease, 2 patients due to intercurrent diseases and one patient due to iatrogenic cause. At the last observation date, 15 patients remained alive, and 2 patients were lost to follow up.

**Figure 3:**
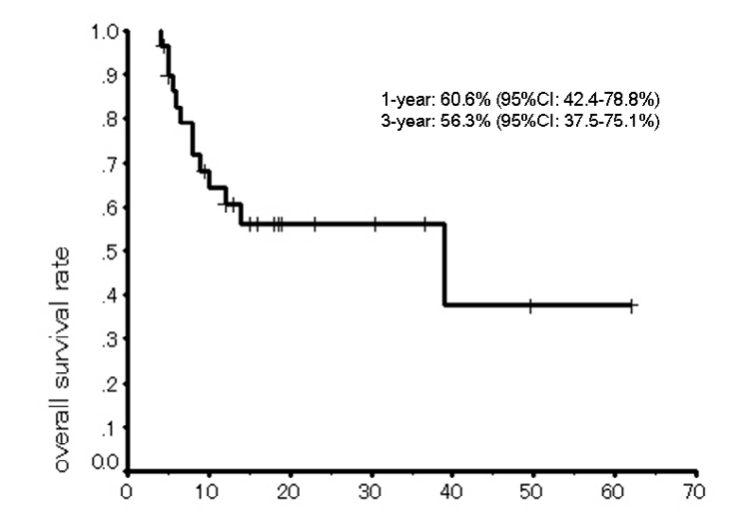
Overall survival of patients with postoperative locoregional recurrent esophageal cancer/(Kaplan-Meier method).

The 1-year and 3-year overall survival rates were 60.6% (95%CI = 42.4–78.8) and 56.3% (95%CI = 37.5–75.1), respectively, with a median survival period of 39.0 months (95% CI = 0.0–82.3) (Fig. 3). Overall response rate, including complete responses in 4 patients and partial responses in 18 patients, was 73.3% (Table 2). There was not correlation between tumor response and site of recurrence or between overall survival rate and site of recurrence.

**Figure 4:**
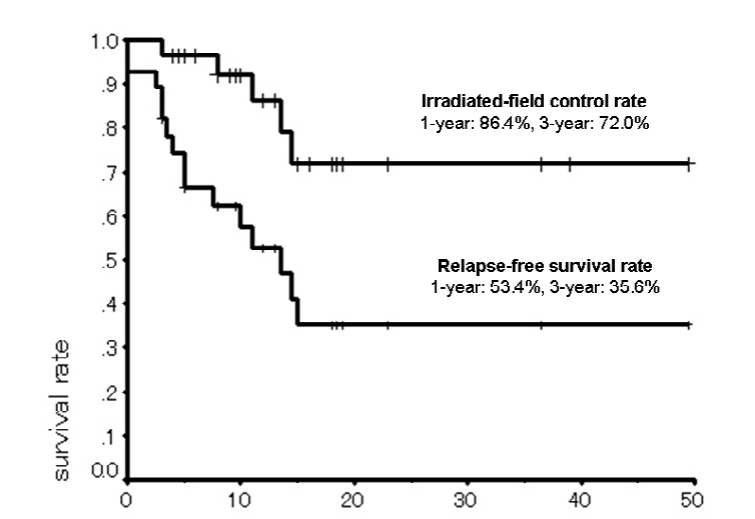
Relapse-free survival and irradiated-field control rates/(Kaplan-Meier method).

The 1-year and 3-year relapse-free survival rates were 53.4% and 35.6%, respectively, and the 1-year and 3-year irradiated-field control rates were 86.4% and 72%, respectively (Fig. 4).

As the major toxicity in the acute phase, grade 3 leukocytopenia was observed in 9 (30%) of the patients. However, grade 4 leukocytopenia was not observed in any of the patients. Thrombocytopenia of grade 3 or higher was observed in one patient, diarrhea of grade 3 or higher was observed in two patients, and heartburn or mucositis of grade 3 or higher was observed in one patient. These grade 3 hemotoxicities were controllable and transitory, and some patients with grade 3 hemotoxicity were therefore able to complete the regimen without suspension of treatment or reduction of dose in the second cycle of chemotherapy. Grade 1 renal toxicity was observed in one patient, but no patient had renal toxicity of grade 2 or higher. These toxicities were manageable and there was no fatal (grade 5) toxicity in the acute phase (Table 4). However, one patient died 6 months after the protocol due to serious pericardial effusion. There were no patients often than the patient who had grade 3 or higher toxicities in the late phase, although grade 1 or 2 focal pulmonary fibrous change, pericardial effusion and/or pleural effusion were often observed. There was a strong correlation between radiation field (T-shaped or local alone) and acute adverse events (<grade 3 or >grade 3), rate of occurrence of grade 3 or higher adverse events being significantly higher in patients who underwent radiotherapy with a T-shaped field (p = 0.046; Pearson's product moment correlation coefficient = -0.367).

In univariate analysis, the difference between survival rate in performance status (p = 0.033, Exp (B) = 4.599, 95%CI = 1.131–18.696), age (p = 0.034, Exp (B) = 0.909, 95%CI = 0.833–0.993) and recurrent pattern (p = 0.024, EXP (B) = 0.261, 95%CI = 0.081–0.836) were statistically significant (Table 5).

## Discussion

There have been some studies on the effectiveness of radiotherapy (with or without chemotherapy) for treatment of postoperative recurrent esophageal cancer. In those studies, the median survival periods were 7.0~9.5 months and the 1-year survival rates were 28~69% (Table 3) [12-16,25]. The median survival periods in previous studies on the effectiveness of chemotherapy alone for treatment of recurrent esophageal cancer were similar, 5.0~10.5 months [10,17-19]. None of the results were good. In 2001, we also reported the results of a study on the effectiveness of radiotherapy (with or without concurrent chemotherapy) for treatment of postoperative locoregional recurrent esophageal cancer: the median survival period of patients who did not undergo chemotherapy was 7.0 months, the median survival period of patients who underwent chemotherapy (CDDP and 5-FU) was 9.0 months, and the overall 1-year and 3-year survival rates were 33 and 15%, respectively [15]. In 2003, we reported the results of a study on the effectiveness of radiotherapy combined with CDGP + 5-FU in our institution for treatment of 7 patients with postoperative locoregional recurrent esophageal cancer: the 1-year survival rate was 69% (The median survival period could not be calculated.) [16]. Compared with these results, the results of the present study showing overall median survival period of 39.0 months and 1-year survival rate of 60.6% were excellent. Although it seems natural that chemoradiotherapy is more effective than radiotherapy or chemotherapy alone because of their synergistic and/or additive effects, the high rate of completion of the regimen because of less toxicities of CDGP and the high local control rate might have contributed to the better results in our study than those in previous studies on chemoradiotherapy. Although we have no evidence, CDGP or CDGP + 5-FU might have a greater synergy with radiation than CDDP or CDDP + 5-FU. However, we also should consider some biases: for example, the observation period was too short to determine long-term results, patients with performance status of 4 were excluded, patients who had hematogenous metastasis were excluded, all patients had squamous cell carcinoma by pure chance, high dose radiotherapy could be performed easily because of the unused adjuvant nor neoadjuvant therapy in association with initial surgery, and some of the 16 patients who had relapse again were treated with second-line salvage chemotherapy (docetaxel alone, TS-1 alone, or combined with CDDP or CDGP and docetaxel) after relapse again. The median survival period of the 16 patients after relapse again was 9.5 months (95%CI = 2.4–15.6).

However, only 5 of the 30 patients in the present study had relapse again inside the irradiation field. Irradiated-field control rates were 86.4% at 1-year and 72% at 3-year. Eleven of the 30 patients had relapse again in lymph nodes outside the irradiation field or in distant organs. There was no significant difference between survival periods of patients who had relapse again inside the irradiated-field and patients who had no relapse again inside irradiated-field. However, we believe that the high irradiated-field control rate of this protocol contributed to prolongation of survival. Although there was also no significant difference (median survival time; one region vs. multiple regions: 39.0 vs. 6.5 months, p = 0.19), number of recurrent regions also might have no small effect on survival.

Regarding the optimal irradiation field, we have experienced whether we need to irradiate to three-field including the bilateral supraclavicular, mediastinal and celiac regions or just the recurrent region of recurrence. In the present study, a T-shaped field was used in 11 patients. Although there was no significant difference, the median survival period of patients who received irradiation to the recurrent region alone was longer than that of patients who received T-shaped field irradiation (local vs. T-shaped: 39.0 vs. 14.0 months, p = 0.17; log-rank test). It was thought that this difference in the median survival period was due to the fact that a T-shaped field was often used for patients having multiple regional recurrences, although there was no significant correlation. Moreover, the problem of the significantly high rate of adverse events in patients who were treated with a T-shaped field remains, although most of these adverse events were controllable. To our knowledge, there is no report about irradiation fields for postoperative recurrent esophageal cancer. Prospective randomized studies on irradiation fields are needed.

The optimal radiation dose for recurrent esophageal cancer had also not been determined. About 60 Gy of radiotherapy combined with chemotherapy is preferred in Japan. Since TD5/5 (prediction radiation-dose of normal tissue complication probability at 5% within 5 years after radiotherapy) of the stomach is 60 Gy [27] and since one of our patients died of necrosis of the stomach, which had been used for thoracic esophageal substitution, 6 months after the end of 66 Gy radiotherapy, we avoid irradiation to the gastric tube with a total dose of more than 60 Gy. We have no major matter of stomach using 60 Gy/30 fractions/6 weeks radiotherapy. But the tolerance radiation-dose of gastric tube with chemotherapy has not been known. Although patients with primary esophageal cancer in the Intergroup Trial (INT) 0123 (Radiation Therapy Oncology Group (RTOG) 94-05) study were assigned randomly to receive combined-modality therapy consisting of CDDP (75 mg/m^2 ^bolus day 1) + 5-FU (1000 mg/m^2^/24 hours for 4 days) with concurrent 64.8 Gy of radiotherapy or the same chemotherapy schedule but with concurrent 50.4 Gy of radiotherapy, there was no significant difference in median survival, 2-year survival, or local/regional failure and local/regional persistence of disease [26]. Therefore, at present, four cycles of CDDP + 5-FU combined with 50 Gy of radiotherapy is a standard chemoradiotherapy regimen for advanced esophageal cancer in U.S.A.. As for primary esophageal cancer, a total dose 50 Gy radiotherapy with concurrent chemotherapy may be sufficient for recurrent esophageal cancer. It is necessary to investigate prospectively the optimal radiation dose for postoperative locoregional recurrent esophageal cancer.

CDGP has shown less renal toxicity but an anti-tumor effect similar or superior to that of CDDP in some preclinical and clinical studies [20-23]. Although the optimal doses of CDGP and 5-FU with radiotherapy have not been determined, we used doses of the anti-tumor drugs in the present study based the report by Yoshioka et al. [25]. They administered CDGP at a dose of 80 or 100 mg/m^2^/2 hours and 5-FU at a dose of 350 or 500 mg/m^2^/24 hours for 5 days and recommended 100 mg CDGP/m^2 ^and 500 mg 5-FU/m^2^. In the present study, we decided to administer CDGP at a 70% dose of their recommendation with consideration of concurrent radiotherapy.

The major toxicities are listed in Table 4. About major toxicities of this protocol in the acute phase, grade 4 toxicity was observed in only two patients. Grade 3 or higher toxicities of neutropenia, thrombocytopenia and esophagitis occurred in 30%, 3.3% and 3.3% of the patients, respectively. However, these grade 3 toxicities were temporary or controllable, and the protocol was performed in 23 patients without suspension or discontinuation and without reduction in the dose of chemotherapy. Therefore, the rate of completion of this regimen was high (76.7%). Compared to results of some clinical studies on chemoradiotherapy, for example, a phase II study by Ohtsu et al. [28], Japan Clinical Oncology Group Trial (JCOG) 9516 [29], a report by Burmeister et al. [30], RTOG 85-01 [31] and INT 0123 [26], which cause a standard regimen for primary esophageal cancer in U.S.A., the rate of grade 4–5 toxicity in this study was low. The chemoradiotherapy protocol used in this study is therefore feasible and safe. The results of several studies, including the present study, indicate that CDGP + 5-FU is no less safe and effective than CDDP + 5-FU [16,17,24,25]. Extensive prospective randomized studies are needed to compare the effectiveness and safety of radiotherapy combined with CDGP + 5-FU and those of radiotherapy combined with CDDP + 5-FU.

As prognostic factors of postoperative recurrent esophageal cancer, PS, age (worse for younger patients) and recurrent pattern (worse for patients with anastomotic recurrence), which had no correlation with others, were significantly associated with survival in univariate analysis in the present study (Table 5). The reason for the poor prognosis of young patients is not known. Tumors in younger patients may be aggressive, although there was no significant correlation between age and the time interval between surgery and relapse in the present study. The reason for the poor prognosis of patients with anastomic recurrence might be because of the significant correlation with PS and pattern of recurrence (p = 0.002, Pearson's product moment correlation coefficient = -0.539; the patients with anastomic recurrence were worse PS). We previously reported that the interval between surgery and relapse was a prognostic factor of recurrent esophageal cancer [15], but this was not selected as a prognostic factor in univariate analysis in the present study.

## Conclusion

The present protocol of radiotherapy combined with nedaplatin and 5-fluorouracil is a safe and effective salvage treatment for postoperative locoregional recurrent esophageal cancer.

## Competing interests

The author(s) declare that they have no competing interests.

## Authors' contributions

KJ and KN drafted the manuscript. KJ and EN performed statistical analysis. KN, YT and SY participated in the study design and coordination. HM, CT, YO and TS performed the chemoradiotherapy and the follow-up. All the authors have read and approved the final manuscript.

## Pre-publication history

The pre-publication history for this paper can be accessed here:



## Figures and Tables

**Table 1:** Patient characteristics

**Patients**	
**Age**	**years old**
median	64
range	50–72
**Gender**	**Number of patients**
male	29
female	1
Preoperative Stage (UICC* 1997)	**Number of patients**
I	4
II A	2
II B	3
III	17
IV	2
unknown	2
**Site of recurence**	**Number of patients**
supraclavicular lymph node	9
mediastinal lymph node	14
abdominal lymph node	7
local	9
**Performance Status (ECOG†)**	**Number of patients**
0	10
1	15
2	3
3	2
4	0
State at last observation date (August 31, 2005)	**Number of patients**
alive	15
dead	13
unknown	2

* UICC: Union Internationale Contre le Cancer, † ECOG: Eastern Cooperative Oncology Group

**Table 2:** Treatment response (RECIST: Response Evaluation Criteria in Solid Tumors)

**Treatment response**	No.
CR*	4
**PR§**	18
**SD†**	7
**PD#**	1

CR: complete response, §PR: partial response, † SD: stable disease, #PD: progression disease

**Table 3:** Contents and results of radiotherapy (with or without chemotherapy) for postoperative recurrent esophageal cancer. in previous studies. The numbers in parenthesis are a numbers of patients.

**author**	**year**	**No.**	**regimen**	**response rate**	**median survival time**	**1-year survuval rate**
JL Raoul [13]	1995	24	radiotherapy + chemotherapy	65%	-	47.1%
H Yamanaka [25]	1998	17	radiotherapy + CDGP§ + 5-FU#	76.50%	-	-
K Nemoto [15]	2001	33	radiotherapy alone(21) or radiotherapy + CDDP* + 5-FU (12)	-	7 months	33%
Y Nishimura [12]	2003	13	radiotherapy + CDDP + 5-FU	72%	9.5 months	28%
H Shimada [14]	2003	76	chemoradiotheray(47), chemotherapy alone(17), radiotherapy alone(12)	34%	8 months	31%
K Nemoto [16]	2003	7	radiotherapy + CDGP + 5-FU	100%	-	69%

* CDDP: cisplatin, § CDGP: nedaplatin, # 5-FU: 5-fluorouracil

**Table 4:** Major toxicities related to this regimen (the Common Terminology Criteria for Adverse Events: CTCAE v3.0)

Toxicity	Grade 1	Grade 2	Grade 3	Grade 4	Grade 5
neutropenia	16.7%	33.3%	30%	0%	0%
thrombocytopenia	3.3%	0%	0%	3.3%	0%
heartburn or mucositis	50%	3.3%	3.3%	0%	0%
renal toxicity	3.3%	0%	0%	0%	0%
diarrhea	16.7%	20%	3.3%	3.3%	0%

**Table 5:** Prognostic factors. Cox's proportional hazards regression model was used for univariate survival analysis.

factor	group	No.	median survival time	log-rank	univariate
			
			(month)	p	p
performance status	0–1	25	39	0.018	0.033
	2–3	5	8		
age	≥65	12	39	0.017	0.034
	<65	18	10		
preoperative stage (UICC§ 1997)	I – II	9		0.87	0.87
	III – IV	19	39		
number of cycles of chemotherapy	1	5	9	0.50	0.501
	2	25	39		
time interval between surgery and recurrence	>13	14		0.083	0.212
	≤13	16	14		
tumor response (RECIST*)	CR-PR	22	39	0.27	0.277
	SD-PD	8	12		
field	local	19	39	0.17	0.183
	T-shaped	11	14		
relapse again inside irradiated field	+	5		0.57	0.594
	-	25	39		
number of recurrent regions	one	23	39	0.19	0.206
	multiple	7	6.5		
recurrent pattern	local	9	6.0	0.015	0.024
	non-local	21	39.0		

*RECIST: Response Evaluation Criteria in Solid Tumors, §UICC: Union International Contre le Cancer

Three blank columns show that the median survival times could not be calculated.
